# The structural neuroanatomy of music emotion recognition: Evidence from frontotemporal lobar degeneration

**DOI:** 10.1016/j.neuroimage.2011.03.002

**Published:** 2011-06-01

**Authors:** Rohani Omar, Susie M.D. Henley, Jonathan W. Bartlett, Julia C. Hailstone, Elizabeth Gordon, Disa A. Sauter, Chris Frost, Sophie K. Scott, Jason D. Warren

**Affiliations:** a Dementia Research Centre, Institute of Neurology, University College London, UK; b Department of Medical Statistics, London School of Hygiene and Tropical Medicine, London, UK; c Max-Planck-Institute for Psycholinguistics, Nijmegen, The Netherlands; d Institute of Cognitive Neuroscience, University College London, London, UK

**Keywords:** Music, Emotion, Dementia, Frontotemporal, FTLD, VBM

## Abstract

Despite growing clinical and neurobiological interest in the brain mechanisms that process emotion in music, these mechanisms remain incompletely understood. Patients with frontotemporal lobar degeneration (FTLD) frequently exhibit clinical syndromes that illustrate the effects of breakdown in emotional and social functioning. Here we investigated the neuroanatomical substrate for recognition of musical emotion in a cohort of 26 patients with FTLD (16 with behavioural variant frontotemporal dementia, bvFTD, 10 with semantic dementia, SemD) using voxel-based morphometry. On neuropsychological evaluation, patients with FTLD showed deficient recognition of canonical emotions (happiness, sadness, anger and fear) from music as well as faces and voices compared with healthy control subjects. Impaired recognition of emotions from music was specifically associated with grey matter loss in a distributed cerebral network including insula, orbitofrontal cortex, anterior cingulate and medial prefrontal cortex, anterior temporal and more posterior temporal and parietal cortices, amygdala and the subcortical mesolimbic system. This network constitutes an essential brain substrate for recognition of musical emotion that overlaps with brain regions previously implicated in coding emotional value, behavioural context, conceptual knowledge and theory of mind. Musical emotion recognition may probe the interface of these processes, delineating a profile of brain damage that is essential for the abstraction of complex social emotions.

## Research highlights

► Emotion recognition from music is impaired in frontotemporal lobar degeneration. ► This deficit is associated with atrophy in a distributed cerebral network. ► This network includes cortical and mesolimbic areas likely to code social emotions.

## Introduction

Despite much recent interest in the neurobiology of music, the brain mechanisms that are critical for processing emotion in music remain incompletely understood. Music is universal and highly valued for the powerful emotional responses it engenders: indeed, music activates brain circuitry associated with pleasure and reward (Blood and Zatorre, 2001; Brown et al., 2004; Menon and Levitin, 2005; Boso et al., 2006; Koelsch et al., 2006; Mitterschiffthaler et al., 2007; Salimpoor et al., 2011) and musical emotion judgments and brain responses are consistent amongst members of a musical culture (Peretz et al., 1998; Menon and Levitin, 2005). Certain music can specifically induce an intense arousal response in normal listeners (Blood and Zatorre, 2001), and this response is mediated by brain structures such as the amygdala and insula that have been implicated in encoding key dimensions of many other kinds of salient emotional stimuli (Adolphs et al., 1994; Anderson et al., 2000; Calder et al., 2001; Cardinal et al., 2002; Dolan, 2007). Deficits of musical emotion comprehension have been reported following focal damage of these same structures (Griffiths et al., 2004; Gosselin et al., 2007). This is surprising, as the biological relevance of music is less clear than for other kinds of emotional stimuli (Blood and Zatorre, 2001): unlike emotion-laden animate stimuli such as human faces and voices, music is an abstract entity without obvious survival value. Nevertheless, music serves a clear social role in all human cultures, raising the possibility that the processing of musical signals may have certain similarities with the processing of other kinds of complex social and emotional signals. Music engages brain areas involved in the formation of learned associations and representation of value in stimuli, including orbitofrontal cortex (OFC) (Rolls, 2004; Menon and Levitin, 2005; Dolan, 2007), as well as dopaminergic reward circuitry (Salimpoor et al., 2011). This conjunction may be the basis for a biologically relevant role for music that is more or less specific for our species.

Whilst the processing of musical emotion is likely to involve brain mechanisms that are partly shared with mechanisms that process other emotional stimuli, understanding of the emotional content of music may also depend on additional brain mechanisms. These brain mechanisms might abstract emotional information from inanimate signals that are qualitatively different from the animate emotional signals conveyed by facial and vocal expressions. One candidate brain mechanism of this kind might be engaged in ‘theory of mind’ processing: the attribution of mental states to other individuals using emotional and other social cues (Gallagher and Frith, 2003) and based on learned social ‘rules’ and concepts (Ross and Olson, 2010), including those embodied in music (Steinbeis and Koelsch, 2009). Brain areas that mediate such processes include medial prefrontal and anterior temporal lobe cortices (Saxe et al., 2004; Gallagher and Frith, 2003; Carrington and Bailey, 2009). Neural mechanisms of musical emotion have potentially far-reaching implications for understanding how the brain codes emotional value, and how emotional signals acquire meaning.

Although the brain basis of music emotion processing has been studied using functional imaging techniques in healthy subjects (e.g., Blood and Zatorre, 2001; Koelsch et al., 2006), to establish essential neural substrates requires alternative approaches that address the effect of strategic brain damage (Griffiths et al., 2004; Stewart et al., 2006). Within the wide spectrum of disease processes that can potentially affect cognition, there is an important distinction between focal and neurodegenerative pathologies. Whereas focal brain lesions tend to occur stochastically or determined by blood supply or physical factors, neurodegenerative diseases strike distributed but functionally connected brain networks (Seeley et al., 2009; Zhou et al., 2010): though highly heterogeneous in their expression in individual patients, these diseases have relatively predictable and selective network signatures at group level. Furthermore, the networks targeted by neurodegenerative pathologies show intrinsic functional connectivity in the healthy brain (Seeley et al., 2009), providing a compelling rationale for studying neurodegenerative network breakdown in order to draw inferences about brain organisation in health as well as disease. The potential of neurodegenerative disease as a complementary cognitive lesion model was first appreciated in the case of the progressive aphasias and the language system (Hillis, 2007). However, the model is no less relevant for emotion and music processing, as exemplified by the group of focal non-Alzheimer dementias collectively known as the frontotemporal lobar degenerations (FTLD).

The diseases in the FTLD spectrum are characterised by brain atrophy chiefly affecting the frontal and temporal lobes, and many patients present with derangements of complex social and emotional behaviour (Seeley et al., 2009; Schroeter et al., 2008; Zhou et al., 2010). Impaired emotion processing in FTLD has been documented for facial expressions (Snowden et al., 2001; Rosen et al., 2002,2004; Fernandez-Duque and Black, 2005; Kessels et al., 2007), voices (Keane et al., 2002; Snowden et al., 2008), and music (Matthews et al., 2009; Omar et al., 2010). Deficits of theory of mind processing are also well documented in FTLD (Adenzato et al., 2010). Neurobiologically, FTLD presents a unique ‘experiment of nature’. The canonical FTLD syndromes of behavioural variant frontotemporal dementia (bvFTD) and semantic dementia (SemD) are both associated with clinically significant emotional dysfunction (Snowden et al., 2001) and these syndromes are underpinned by dissociable large-scale neural networks mediating different aspects of emotion and higher order sensory processing. bvFTD has been linked with an anterior peri-allocortical ‘salience network’ mediating responses to emotional stimuli, whilst SemD has been linked with a fronto-temporal network mediating conceptual knowledge about sensory objects (Seeley et al., 2007,2009; Zhou et al., 2010). Moreover, FTLD is a relatively common cause of dementia, enabling group-level neuroanatomical correlation. From a clinical perspective, investigation of musical emotion processing and its cerebral associations in FTLD has the potential to improve our understanding of disease phenomenology and pathophysiology, and more specifically, the influences that modulate intrinsic network connectivity in the working brain.

The key objective of this study was to investigate critical neuroanatomical associations of emotion recognition from music using FTLD as a disease model of brain network breakdown. The processing of emotion in music is likely to be a hierarchical and multi-component process (Juslin and Laukka, 2003; Juslin and Västfjäll, 2008; Zentner et al., 2008; Koelsch, 2010) and it is therefore important at the outset to define what is being assessed. In this study we were interested chiefly in overt recognition of musical emotions, as indexed by patients' ability to categorise the dominant emotional characteristics expressed by a particular musical piece. We used a behavioural paradigm comparable to that used previously to assess emotion recognition in other modalities (fixed-alternative, forced-choice verbal labelling of the expressed emotion) in order to compare performance on recognition of canonical emotions as represented in music with the same emotions in human facial expressions and nonverbal vocal sounds. Anatomical associations of emotion recognition performance were assessed using voxel-based morphometry (VBM). Because music requires the abstraction of emotional content from inanimate cues, we hypothesised that emotion recognition from music in FTLD is vulnerable to the effects of damage involving distributed brain circuitry for representing and evaluating the emotional content of stimuli. Specifically, we hypothesised that the brain mechanisms mediating musical emotion recognition performance in FTLD would recruit areas previously implicated in processing valence, salience and subjective states associated with other kinds of emotion-laden stimuli, including mesial temporal structures, insula and their connections in the mesolimbic system. In addition, we hypothesised that recognition of emotion in music would place particular demands on brain mechanisms involved in analysis and evaluation of the emotional content of complex social signals, including OFC, medial prefrontal and anterior temporal cortex. Following the emerging neural network paradigm of neurodegenerative disease (Seeley et al., 2009; Zhou et al., 2010), we predicted joint involvement of the emotional salience processing network (including anterior cingulate, insula and frontal pole) and the conceptual processing network (including temporal pole, amygdala, orbitofrontal cortex and ventral striatum) previously linked with canonical syndromes of FTLD.

## Methods

### Subjects

Twenty-six patients (18 male, 24 right-handed, mean (SD) age 63.8 (8.4) years) fulfilling consensus criteria for a diagnosis of FTLD (Neary et al., 1998) were recruited from the tertiary-level Specialist Cognitive Disorders Clinic at the National Hospital for Neurology and Neurosurgery, London, United Kingdom. Twenty-one healthy control subjects with no history of neurological, psychiatric or otological illness and matched with the patient group for age and educational background also participated. The patient cohort comprised two canonical FTLD subtypes: 16 patients had bvFTD, characterised by profound personality and behavioural change with frontal and temporal lobe atrophy on brain magnetic resonance imaging (MRI); and 10 patients had SemD, characterised by breakdown of verbal and nonverbal conceptual knowledge systems with asymmetric, predominantly left-sided temporal lobe atrophy on MRI. These FTLD subgroups were targeted on account of their known propensity to exhibit clinical deficits of emotion recognition; all cases included in this series had typical clinical and radiological profiles of bvFTD or SemD, as previously described (Edwards-Lee et al., 1997; Chan et al., 2001; Liu et al.*,* 2004). No patients had a history of deafness or other hearing abnormalities. In all patients, the clinical syndrome was further characterised with an assessment of general neuropsychological functions. Most subjects had fewer than two years formal music training, corresponding to the ‘least trained’ (novice and non-musician) category of musical experience described by Halpern et al. (1995): one of the patients was a professional musician, and two control subjects had five years of piano lessons in childhood. Subject demographic characteristics and background neuropsychological results are summarised in Table 1. Informed consent was obtained in each case and the study was approved by the local research ethics committee in accord with Declaration of Helsinki guidelines.

### Behavioural assessment of emotion recognition

Recognition of four emotions (happiness, sadness, anger, and fear) from music, facial expressions and nonverbal vocal sounds was assessed using a procedure in which subjects were required to match each target stimulus with the most appropriate verbal emotion label in a four-alternative-forced-choice paradigm. The target emotions chosen represent four of the six canonical emotions in the original set of emotional facial expressions created by Ekman and Friesen (1976); surprise and disgust were excluded due to the difficulty of creating musical equivalents for these. The four target emotions were included in order to sample the emotion spectrum in each modality using a uniform paradigm; however, this study was designed principally to assess modality-level effects rather than individual emotion effects. Ten examples of each target emotion (i.e., a total of 40 trials) were presented in each modality. The music stimuli were short (approx. 11 s) non-vocal (orchestral and chamber) excerpts drawn from the Western classical canon and film scores; the creation of these music stimuli and administration of the music emotion recognition battery have been described previously (Omar et al., 2010). Facial expression stimuli were derived from the Ekman and Friesen (1976) set; vocal stimuli were derived from a set developed by Sauter et al. (2010). Details of the behavioural protocol and stimuli are provided in Supplementary Material on-line.

Behavioural data for each FTLD subgroup, for the combined FTLD group and for the healthy control group were analysed using Stata©. For each emotion modality, we found the mean total recognition score (/40) and 95% bias-corrected bootstrap confidence intervals (100,000 bootstrap samples). Interpretation of the behavioural findings in the FTLD group requires that control performance is taken into account: emotion recognition performance is unlikely to be uniform between modalities, even in healthy subjects, however there are no widely accepted metrics for comparing recognition scores across modalities. In order to provide such a metric here, we assessed how well emotion modalities and emotion:modality combinations were able to discriminate FTLD patients from healthy controls, using receiver operating characteristic (ROC) curves; the discriminatory ability of each metric was quantified using the area under the curve (AUC). The AUC is the probability that, in a randomly selected patient/control pair, the patient has a lower emotion recognition score than the control (Hanley and McNeil, 1982); perfect discrimination between patient and control groups would correspond to an AUC of 1, whilst the same distribution of scores in patients and controls would correspond to an AUC of 0.5. To allow for any differences in demographics between subject groups, we calculated covariate-adjusted AUCs (Janes and Pepe, 2008; Janes et al., 2009), using the linear adjustment method with covariates of age, gender, and years of education. We assessed covariate adjusted AUCs for discriminating between the bvFTD subgroup and controls, between the SemD subgroup and controls, and between FTLD patients (ignoring subtype) and controls. We similarly assessed the utility of emotion recognition scores in discriminating between bvFTD and SemD subgroups using adjusted AUCs (initially assuming for purposes of the analysis that SemD subjects would have higher scores) with covariates of age, gender and years of education. Bias-corrected bootstrap confidence intervals were found using 100,000 bootstrap samples. Differences between AUCs were assessed using a z-test with the bootstrap-estimated standard error. Associations between emotion recognition in each modality and a measure of executive function (Trail Making part B scaled score) were assessed using logistic regression models for the individual item response in each modality (emotion correctly recognised = 1), with random subject and item effects and Trails score and emotion as fixed effects.

### Brain image acquisition and analysis

#### Image acquisition

MR brain images were acquired in all FTLD patients at the time of behavioural testing, on the same 1.5T GE Signa scanner (General Electric, Milwaukee, WI) using an IR-prepared fast SPGR sequence (TE = 5 ms, TR = 12 ms, TI = 650 ms). T1-weighted volumetric images were obtained with a 24 cm field of view and 256 × 256 matrix to provide 124 contiguous 1.5-mm-thick slices in the coronal plane.

#### Image analysis

Brain images were processed using MATLAB 7.0® and SPM2® (http://www.fil.ion.ucl.ac.uk/spm/). Voxel-based morphometry (VBM) was performed using a modified version of an optimised method (Good et al., 2001; Henley et al., 2008; Ridgway et al., 2009). The native space study images were affine-registered using the standard SPM2 T1 template, and initial grey matter segmentation was performed. Normalisation parameters were estimated for warping these grey matter segments onto the SPM2 grey matter template, and these normalisation parameters were then used to warp the original native space images. Segmentation of the normalised images into grey matter was then performed and these segmentations modulated with the volume changes from the normalisation step. Each grey matter segment had non-brain tissue removed according to a brain mask derived from the corresponding original image using semi-automated segmentation software (Freeborough et al., 1997). The images were then smoothed with an isotropic Gaussian kernel of 8 mm FWHM.

Linear regression was used to examine voxel-wise associations between grey matter volume and emotion recognition performance, modelling voxel intensity as a function of emotion recognition score. Neuroanatomical associations of emotion recognition in the three modalities were assessed in separate design matrices for each modality (separate-modality analysis) and in a combined regression matrix including all three modalities (combined-modalities analysis); the latter analysis was designed to assess associations of emotion recognition in a particular modality after adjusting for any association with other modalities and to directly compare modalities. In the combined-modalities analysis, direct pair-wise contrasts between emotion recognition regressors were assessed for music with respect to each of the other modalities; in addition, in order to identify grey matter associations common to different modalities, a conjunction analysis was run for music with respect to each of the other modalities. Age, gender, total intracranial volume (calculated using a previously described procedure: Whitwell et al., 2001) and disease duration were incorporated as covariates in all design matrices. In addition, in order to assess whether grey matter associations of music emotion recognition were modulated by general executive performance, Trails score was also incorporated as a covariate of music emotion recognition score in a separate design matrix. After model estimation an explicit mask was applied using a masking strategy that excluded any voxels for which > 30% of images had intensity value < 0.05 (i.e., consensus 70%, threshold 0.05): this was motivated by previous evidence that SPM2® default threshold masking may exclude the most severely affected regions from statistical analysis in subjects with marked focal atrophy (Ridgway et al., 2009).

For each model, statistical parametric maps were assessed at two statistical thresholds: at p < 0.05 after false discovery rate correction over the whole brain (Genovese et al., 2002), and at p < 0.05 after small volume correction using anatomical regions based on the *a priori* hypotheses. These anatomical small volumes were derived by manual tracing from the template brain image using MRIcro® (http://www.sph.sc.edu/comd/rorden/mricro.html) and comprised: bilateral OFC (including the orbital surface of both frontal lobes and the lateral orbital gyri below the inferior frontal sulcus bilaterally); right and left insula; and right and left temporal lobes anterior to Heschl's gyrus. The volumes were all drawn entirely manually on the group mean normalized brain image based on the patients' T1-weighted structural MRI scans; the volume boundaries were intentionally generous, to ensure that individual variations in brain anatomy were all fully encompassed, however, all anatomical attributions within these volumes were subsequently checked visually in order to ensure accurate localisation to particular regions within the volume. In the conjunction analysis, non-orthogonality between the regressors was assumed and a conjoint conjunction threshold was applied (p < 0.001 for each of the component regressors).

## Results

### Behavioural findings

The performance of the FTLD patients and healthy control subjects on emotion recognition tests in different modalities is summarised in Table 2 (detailed behavioural data including mean recognition scores for individual emotion:modality:combinations are provided in Supplementary Table 2 on-line). Overall, both patient subgroups and healthy control subjects scored highest for emotion recognition from faces, followed by voices, and music. AUC analyses comparing the bvFTD and SemD subgroups separately with healthy controls (Table 2) generally showed similar results to those for the combined FTLD group (detailed AUC data are presented in Supplementary Table 3 on-line). Further when the disease subgroups were directly compared, none of the estimated AUCs differed significantly from 0.5: i.e., there was no evidence that emotion recognition performance differed between bvFTD and SemD. We therefore predominantly focus on AUC results for the combined FTLD group versus healthy controls.

**Fig. 1:**
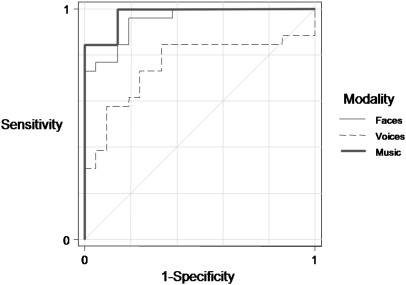
Comparison of emotion recognition in different modalities: prediction of disease by emotion recognition performance. The covariate (age, gender, and years of education) adjusted ROC curves use total emotion recognition scores (/40) in each modality to discriminate between FTLD patients (ignoring subtype) and controls.

Fig. 1 shows covariate (age, gender, and years of education) adjusted ROC curves using emotion recognition performance in each modality to discriminate between FTLD patients (ignoring subtype) and controls. Comparing total music emotion recognition scores between the combined FTLD group and controls, the AUC was 0.98 (95% CI 0.86, 1.00; p < 0.05): i.e., an estimated 98% probability that a randomly selected patient scores lower than a healthy control subject matched for age, gender and education. The AUC for total facial emotion recognition score was similar to music (0.95, 95% CI 0.84, 0.99; p < 0.05). There was no evidence that the music and face emotion modalities differed in their discriminatory ability (p = 0.45). These results indicate that performance on music emotion recognition and facial emotion recognition each indexes a true neuropsychological deficit in the FTLD group. The AUC for total vocal emotion recognition score (0.76, 95% CI 0.58, 0.91; p < 0.05) was statistically significantly greater than 0.5, indicating that vocal emotion recognition performance also discriminates FTLD patients from controls. However this discriminatory power was significantly lower than the discriminatory power of emotion recognition from music (p = 0.009) and faces (p = 0.02). For the comparison between the bvFTD subgroup and controls the AUC for total vocal emotion recognition score was not formally statistically significant (0.71, 95% CI 0.46, 0.90): nevertheless it was directionally consistent with that for the comparison between the SemD subgroup and controls and when the two subgroups were directly compared the AUC did not differ significantly from 0.5. There was a significant association between executive performance (Trails score) and emotion recognition from music (odds ratio 1.090; 95% confidence intervals (1.032, 1.151)) and from voices (odds ratio 1.121; confidence intervals (1.036, 1.212)).

### Neuroanatomical associations

**Fig. 2:**
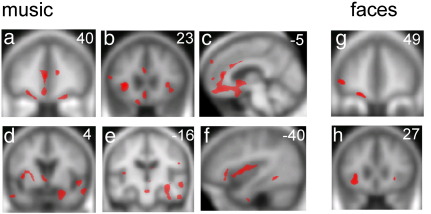
Statistical parametric maps (SPMs) of grey matter loss associated with impaired emotion recognition from music and faces in FTLD. Maps are based on separate-modality regression analyses (see Methods). SPMs are presented on sections of the mean normalised T1-weighted structural brain image in MNI stereotactic space; the left hemisphere is on the left and slice coordinates in mm are shown. For music, SPMs are thresholded at p < 0.05 FDR corrected for multiple comparisons over the whole brain volume; for faces, SPMs are thresholded at p < 0.001 uncorrected for display purposes. The SPMs for music and face emotion recognition show common regional grey matter associations in anterior insula (b,h) and lateral orbitofrontal cortex (a,g). In addition, the SPM for music emotion recognition shows grey matter associations in anterior cingulate (b,c), medial prefrontal cortex (a,c), anterior and superior temporal cortices (d,e,f), fusiform and parahippocampal gyri (e,f), more posterior parietal cortices (e), limbic areas including amygdala and hippocampus (d,e), and other subcortical structures including nucleus accumbens (c,d) and ventral tegmentum (e).

As the behavioural profiles of the bvFTD and SemD subgroups were very similar, we here report the results of the VBM analysis for the combined FTLD cohort. Statistical parametric maps of grey matter regions associated with emotion recognition performance from music and facial expressions are shown in Fig. 2; local maxima of grey matter loss are summarised in Table 3. Considering first the separate-modality analyses, recognition of emotion from music was positively associated with grey matter in an extensive bilateral cerebral network including insula, anterior cingulate, OFC and medial prefrontal (anterior paracingulate) cortex, dorsal prefrontal, inferior frontal, anterior and superior temporal cortices, fusiform and parahippocampal gyri, more posterior parietal cortices, limbic areas including amygdala and hippocampus, and other subcortical structures including nucleus accumbens and ventral tegmentum (all at significance threshold p < 0.05 corrected for multiple comparisons over the whole brain). Covarying for a general executive measure (Trails score) produced a very similar profile of grey matter associations (Supplementary Fig. 1 on-line). Recognition of emotions from facial expressions was positively associated with grey matter in left lateral OFC and bilateral insula (p < 0.05 corrected for anatomical small volumes of interest). No anatomical associations of emotion recognition from voices were identified at the prescribed threshold (p < 0.05 corrected for anatomical small volumes of interest). When emotion modalities were compared in a combined regression analysis incorporating all modalities, grey matter associations of emotion recognition from music were very similar to those revealed by the separate music-only unadjusted regression analysis (at threshold p < 0.05 corrected for anatomical small volumes of interest). In the combined-modalities analysis, no grey matter associations of facial or vocal emotion recognition were identified at the prescribed threshold.

Although certain grey matter regions were similarly associated with emotion recognition from music and faces (Table 3), no voxels were found to be common to two or more modalities in a conjunction analysis (conjoint conjunction threshold p < 0.001 uncorrected). In a direct contrast between music and vocal emotion regressors in the combined-modalities analysis, a significantly stronger association with emotion recognition from music versus voices was identified in a bilateral cortical network including lateral OFC, medial prefrontal cortex and insula (all p < 0.05 corrected for anatomical small volumes of interest; local maxima in Table 3). No grey matter areas showed evidence of a significantly stronger (or weaker) association with emotion recognition from music contrasted with faces.

## Discussion

Here we have demonstrated a grey matter profile positively associated with music emotion recognition (i.e., a profile of brain atrophy associated with deficient music emotion recognition) in patients with FTLD. This grey matter profile included extensive, bilateral cerebral areas including insula, OFC, anterior cingulate and medial prefrontal cortex, anterior temporal and more posterior temporal and parietal cortices, amygdala and other limbic structures, and striatum. Music emotion recognition performance was a sensitive and specific marker of brain damage in FTLD (i.e., a predictor of disease) relative to healthy control subjects. When emotion recognition modalities were compared directly, the anatomical pattern of brain damage associated with deficient recognition of emotion in music in FTLD was at least partly independent of recognition performance in other emotion channels (i.e., the association for music was significant after covarying for emotion recognition performance from faces and voices), and significantly stronger than the association with emotion recognition in another auditory modality (voices). Further, these anatomical associations of music recognition were not simply dependent on disease duration (an index of overall disease-associated brain damage) or general executive performance. The neuroanatomical profile observed in this disease group is therefore likely to illustrate an essential brain substrate for emotion recognition in music. In line with our prior anatomical hypotheses, this anatomical profile comprised a distributed cerebral network: indeed, the present findings provide one of the more complete delineations of the entire network supporting music emotion processing in the human brain, and are strikingly convergent with previous network-level analyses in healthy subjects during active processing of musical emotion (Menon and Levitin, 2005). We now consider the components of this network in detail.

As we anticipated, the cerebral associations of music emotion recognition included areas previously implicated in processing certain dimensions of emotion across a range of emotional stimuli. These included areas involved in processing emotional valence and intensity (amygdala, striatum: Adolphs et al., 1994; Anderson et al., 2000; Calder et al., 2001; Blood and Zatorre, 2001; Cardinal et al., 2002; Dolan, 2007; Gosselin et al., 2007; Mitterschiffthaler et al., 2007), ‘reward’ (ventral striatum: Blood and Zatorre, 2001; Cardinal et al., 2002; Brown et al., 2004; Menon and Levitin, 2005; Koelsch et al., 2006; Mitterschiffthaler et al., 2007; Suzuki et al., 2008), coupling of subjective feeling states and autonomic responses (insula: Calder et al., 2001; Blood and Zatorre, 2001; Molnar-Szakacs and Overy, 2006; Critchley, 2009) and representation of stimulus value (OFC: Rolls, 2004; Menon and Levitin, 2005; Dolan, 2007) from music as well as facial expressions and other sensory stimuli. In the present study, anterior insula and lateral OFC damage was associated with impaired emotion recognition from both music and faces, consistent with a generic role for these areas in the analysis, representation and contextual evaluation of emotional signals. Amygdala damage was associated with impaired emotion recognition only from music: we speculate that this might reflect greater dependence on subjective arousal responses for coding musical emotion compared with the other stimuli used in this study (Dolan, 2007). This factor may also account for anterior cingulate gyrus involvement in the music condition (Mitterschiffthaler et al., 2007).

Music emotion recognition performance was associated with a number of other brain areas not identified with facial or vocal emotion recognition. The behavioural data here suggest that patients with FTLD had comparable deficits of music and face emotion recognition, based on the comparable power of a deficit in either modality to detect the presence of disease in relation to the performance of healthy subjects (Fig. 1), and similar variance of music and facial expression recognition scores across the FTLD group. We therefore argue that the more extensive neuroanatomical associations of musical emotion recognition here reflect additional processes that are particularly associated with processing emotions from music (and perhaps less strongly associated with emotion recognition via other modalities). These music-associated brain areas included medial prefrontal (anterior paracingulate) cortex and antero-mesial temporal lobes and the superior temporal sulcus, previously implicated in evaluating diverse emotional stimuli and others' mental states based on conceptual and autobiographical learning and theory of mind processes (Saxe et al., 2004; Gallagher and Frith, 2003; Carrington and Bailey, 2009; Steinbeis and Koelsch, 2009; Ross and Olson, 2010). These neuroanatomical findings corroborate previous evidence in healthy individuals indicating that music is potentially both highly engaging for the human limbic system (Blood and Zatorre, 2001) and a rich source of semantic and autobiographical associations that interact with emotion judgments (Eldar et al., 2007; Eschrich et al., 2008). Whilst the concept of meaning in music is problematic, there is an important sense in which the ‘meaning’ of a piece of music is the emotional message it conveys, which must be actively decoded by the brain based partly on associations learned by exposure to a musical culture (Peretz et al., 1998; Juslin and Västfjäll, 2008) and past experience of the particular musical piece (Eschrich et al., 2008), as well as transcultural factors (Fritz et al., 2009). It is noteworthy that the bvFTD subgroup in this study exhibited a deficit of theory of mind (Table 1) as indexed by the Reading the Mind in the Eyes test (Baron-Cohen et al., 2001; this test is not suitable for patients with SemD as it requires relatively sophisticated verbal comprehension). The neuroanatomical findings in this patient population provide circumstantial evidence for involvement of theory of mind processing in the interpretation of musical emotions. Since the musical pieces used here were all nonvocal ensemble (mainly orchestral) excerpts, it is unlikely the stimuli conveyed a strong sense of individual human agency. Rather, the findings suggest that recognition of emotion in music may entail attribution of a ‘mental state’ to an abstract stimulus. This is consistent with fMRI evidence for mental state attribution to musical pieces by healthy individuals (Steinbeis and Koelsch, 2009).

Previous anatomical and functional evidence in both healthy and disease populations suggests that the disparate brain areas identified here as associated with musical emotion recognition are linked via a distributed brain network or networks. Anatomically, the key structures (amygdala, antero-mesial temporal lobes, insula, striatum, anterior cingulate, OFC and prefrontal cortex) are densely and reciprocally interconnected (Cardinal et al., 2002; Rolls, 2004; Brown et al., 2004; Menon and Levitin, 2005; Gosselin et al., 2006; Dolan, 2007; Schroeter et al., 2008; Seeley et al., 2006,2009). Integrity of this network may be maintained in part by long projection spindle neurons (of Von Economo) concentrated at anatomical hubs including anterior cingulate, insula and prefrontal cortex (Seeley et al., 2006, 2009). Functionally, the components of this putative network have frequently been observed to be coactivated during the processing of emotions in music and other stimuli (Blood and Zatorre, 2001; Menon and Levitin, 2005; Baumgartner et al., 2006; Eldar et al., 2007; Mitterschiffthaler et al., 2007; Carrington and Bailey, 2009; Steinbeis and Koelsch, 2009), and enhanced effective connectivity between mesolimbic and cortical components of the network during music listening has been demonstrated (Menon and Levitin, 2005). Key components of the mesolimbic (ventral tegmental area, nucleus accumbens, amygdala, and hippocampus) and mesocortical (OFC, medial prefrontal cortex) dopaminergic systems were identified in the present study.

The network identified in this and in previous studies could potentially have a generic role in linking brain mechanisms for assigning emotional value (in music and other stimuli) with mechanisms that assess the behavioural context and relevance of the stimulus in relation to conceptual knowledge, memories and other sensory signals. This interpretation fits with involvement of the anterior structures previously implicated in processing emotionally salient stimuli (Seeley et al., 2006, 2009). The present results underscore the involvement of the phylogenetically ancient dopaminergic mesolimbic brain reward system during music processing (Salimpoor et al., 2011), and further suggest that this involvement is not merely epiphenomenal but required for comprehension of the emotional content of music, as previously forecast (Menon and Levitin, 2005). The cortical components of the network may be loaded particularly where cognitive processing demands are high (for example when labelling specific musical emotions, as in the present study). Dependence on interacting frontal and temporal lobe mechanisms for music emotion recognition would be consistent with the similar behavioural performance of the bvFTD (frontotemporal atrophy) and SemD (temporal atrophy) groups here. The profile of network damage we have identified subsumes previous lesion studies demonstrating that defects of emotion recognition in music may result from focal insults involving anterior and mesial temporal lobes, prefrontal cortex, insula and parieto-temporal cortices (Griffiths et al., 2004; Gosselin et al., 2005, 2006, 2007; Stewart et al., 2006; Johnsen et al., 2009). We do not of course argue that the network mediating music emotion recognition as delineated here necessarily or indeed usually operates en bloc. Indeed, the areas identified here could constitute at least two functionally distinct networks, a mesolimbic network involved in assigning behavioural value to music and a cortical network involved in processing this information in the context of past experience, intimately linked by hub structures including the anterior cingulate and insula. This issue of network differentiation is importantly informed by recent evidence concerning the network basis for neurodegenerative disease (Seeley et al., 2009; Zhou et al., 2010). The neuroanatomical associations of music emotion recognition here overlap with both the anterior peri-allocortical salience network previously linked with bvFTD and the temporal pole-subgenual-ventral striatum-amygdala network previously linked with SemD (Zhou et al., 2010). Whilst the intrinsic connectivity profiles and syndromic associations of these networks have previously been shown clearly to be dissociable, the interactions of the networks during cognitive processing remain to be fully explored. Correlation with behavioural performance as in the present study offers a potential avenue to assess network interactions. The extent and nature of network differentiation and modulation by behavioural tasks are key issues for future work.

From a clinical perspective, the present findings corroborate an extensive literature demonstrating that patients with FTLD have deficits processing emotion in various modalities. It has previously been observed that processing of emotion in music may be relatively resistant to brain damage (Peretz et al., 1998): in conjunction with previous evidence (Omar et al., 2010), the present findings suggest a qualification of this conclusion. Processing of musical emotions has been shown to be spared in Alzheimer's disease (Drapeau et al., 2009; Gagnon et al., 2009; Omar et al., 2010), suggesting that the deficit identified here is not a universal accompaniment of neurodegenerative disease but may be relatively specific to certain degenerative pathologies: notably, those in the FTLD spectrum.

This study has several limitations that suggest important directions for future work. It is likely that musical emotion is processed hierarchically: more ‘primitive’ attributes (such as dissonance/consonance, unpleasant/pleasant) and generic emotional responses to highly familiar music (e.g., Matthews et al., 2009) may be potentially more resistant to the effects of brain damage than specific emotion labelling (as was required here). Related to this issue, it will be important to assess measures of musical perceptual function and emotional arousal in target clinical conditions such as FTLD; these factors are likely to impact on emotion judgments in music. More fundamentally, it remains unclear to what extent music can instantiate simple emotion categories such as those represented in canonical facial expressions and how far musical emotions can be categorised verbally (Zentner et al., 2008). An important rationale for this study was to compare processing of emotions in music with analogous emotions in other modalities. However, whilst there is evidence that the taxonomy of emotions in music partly converges with other emotion modalities, the repertoire of music-specific emotions appears to be wide (Zentner et al., 2008): this discrepancy should be explored in future studies. A further factor that may have confounded comparisons between music and other emotion modalities in this and much previous work is the use of more or less familiar musical examples alongside novel stimuli in other sensory channels. Ideally, musical emotion recognition should be assessed using novel musical pieces, in order to assess the extent to which the involvement of brain memory systems in the antero-mesial temporal lobes and beyond (as demonstrated here) reflects the processing of familiarity rather than musical emotion per se. Finally, the chief interest in this study was modality-specific anatomical associations of emotion processing; however, in future work it will be important to address brain substrates for processing particular emotions independently of modality, and interactions between emotion and modality, which the present study was under-powered to detect.

It has been argued elsewhere that music provides a substrate for integrating emotional states with complex social behaviours (Blood and Zatorre, 2001; Molnar-Szakacs and Overy, 2006; Koelsch, 2010). The present study supports this formulation. The processing of emotion in music may act as a model system for the abstraction of emotions in complex real-life social situations and for the breakdown of emotional understanding in particular disease states. This interpretation would be consistent with clinical evidence: whereas FTLD is characterised by impaired comprehension both of social signals and musical emotion, both these capacities are initially retained in Alzheimer's disease (Drapeau et al., 2009; Omar et al., 2010). A capacity to capitalise on past emotional experience encapsulated in music would require interactions between musical emotion and mnestic processing, consistent with present and previous evidence (Juslin and Laukka, 2003; Eldar et al., 2007; Juslin and Västfjäll, 2008; Eschrich et al., 2008; Koelsch, 2010). Recent insights into the organisation of large-scale brain networks provide a framework for addressing these issues (Seeley et al., 2007,2009; Zhou et al., 2010), whilst the distinct network profiles of different dementia diseases (for example, sparing of the salience network in Alzheimer's disease) predict differential patterns of performance in the analysis of musical emotion. Future work should pursue the complementary avenues of functional imaging of the healthy brain and analysis of music processing in other neurodegenerative diseases (for example, Huntington's disease) with defective emotion encoding.

## Figures and Tables

**Table 1:** Subject demographics and scores on general neuropsychological assessment.

	FTLD cases		Controls
bvFTD	SemD
(n = 16)	(n = 10)	(n = 21)
Age	64.7 (8.0)	62.4 (8.8)	67.0 (8.8)
M:F	15:1	3:7	10:11
Years of education	14.1 (3.5)	12.5 (2.4)	13.4 (3.6)
Years of disease duration	6.9 (4.1)	4.6 (1.6)	n/a
Mini-mental state examination score^1^	**26.9 (3.9)**	**24.2 (3.5)**	29.5 (0.7)^d^
Ravens advanced matrices^2⁎^	**9.2 (3.6)**^a^	12.9 (3.6)^b^	13.8 (1.7)
Camden pictorial memory^3^ (/30)	**26.7 (4.7)**^c^	26.8 (5.3)^b^	29.5 (0.7)^d^
Benton facial recognition^4^ (/54)	45.4 (3.8)	46.5 (4.2)	47.2 (3.1)^d^
Famous faces^5^ (/12)	**10.7 (1.9)**	**7.3 (4.5)**	11.9 (0.3)^d^
Synonyms comprehension^6^ (/25)	**20.2 (3.4)**^c^	**16.4 (5.8)**	23.6 (1.4)^d^
Reading the mind in the eyes^7^ (/36)	**17.8 (6.7)**	n/a	24.4 (4.9)^d^
Trail-making test B^8^ (scaled score)	**7.4 (4.7)**	**8.0 (3.3)**	12.0 (2.4)^d^

Mean (SD) values are shown. a, available for 15 bvFTD patients; b, available for 8 SemD patients; c, available for 14 bvFTD patients; d, available for 10 control subjects; *scaled scores; **bold**, significantly inferior to controls (p < 0.05); bvFTD, behavioural variant frontotemporal dementia; FTLD, frontotemporal lobar degeneration; n/a, not available; SemD, semantic dementia. 1 Folstein MF et al., J Psychiatr Res 1975; 12:189–198; 2 Raven J San Antonio, TX: Harcourt Assessment, 2003; 3 Warrington EK, Psychology Press, 1996; 4 Benton AL et al*.* Oxford University Press, 1983; 5 Warrington EK, James M. 1967. Cortex 1967; 3: 317–326; 6 Warrington EK et al*.* Neuropsychol Rehab 1998; 8: 143–154; 7 Baron-Cohen et al*.* J Child Psychiatry 2001 - this test was not administered to patients with SemD, in order to avoid potentially confounding effects from verbal comprehension impairment; 8 Reitan RM, Indiana University Press, 1958.

**Table 2:** Summary of behavioural findings: mean raw scores for healthy control, bvFTD and SemD groups and ROC analysis of inter-group comparisons for emotion recognition performance in different modalities.

Modality	Mean total raw score /40 (SD) (95% CI for mean)			Differences between groups: expressed as areas under the adjusted* ROC curves for discriminating between groups (95% CI)			
Controls n = 21	bvFTD n = 16	SemD n = 10	bvFTD vs controls	SemD vs controls	SemD vs bvFTD	all FTLD vs controls
Music	32.9 (2.63)	21.8 (5.55)	21.2 (6.03)	**0.98**	**0.97**	0.47	**0.98**
	(31.8, 34.0)	(19.2, 24.4)	(17.6, 24.7)	**(0.78, 1)**	**(0.83, 1)**	(0.22, 0.73)	**(0.86, 1)**
Faces	37.6 (1.40)	32.3 (4.29)	32.5 (5.87)	**0.98**	**0.90**	0.61	**0.95**
	(37.0, 38.1)	(30.2, 34.3)	(28.5, 35.4)	**(0.87, 1)**	**(0.69, 1)**	(0.36, 0.87)	**(0.84, 0.99)**
Voices	35.0 (3.26)	29.7 (5.85)	29.0 (8.21)	0.71	**0.84**	0.35	**0.76**
	(33.4, 36.1)	(26.7, 32.3)	(23.8, 33.4)	(0.46, 0.90)	**(0.60, 0.99)**	(0.14, 0.62)	**(0.58, 0.91)**

*Areas adjusted for age, gender, and years of education. bvFTD, behavioural variant frontotemporal dementia; CI, confidence interval; FTLD, frontotemporal lobar degeneration; SD, standard deviation; SemD, semantic dementia. In the AUC analysis, confidence intervals excluding 0.5 (**bold**) provide evidence (p < 0.05) that the corresponding measure has statistically significant discriminatory power for that comparison.

**Table 3:** Local maxima of grey matter loss associated with impaired emotion recognition in FTLD.

Emotion modality	Brain region		MNI coordinates	Z score
R	L	x, y, z (mm)	
Music*		Anterior insula	− 33 23 3	5.10
		ACC	− 4 39 10	4.81
		Lateral OFC	− 17 15 − 23	4.32
	Amygdala		25 6 − 25	4.27
		FG	− 40 − 50 − 7	4.18
		Temporal pole	− 55 7 − 30	4.18
	ACC		13 40 16	4.13
		Medial OFC	− 2 25 − 12	4.05
	Inferior parietal		55 − 18 26	4.03
		Nucleus accumbens	− 8 9 − 4	4.00
	Anterior insula		31 30 0	3.92
	Hippocampus		37 − 16 − 13	3.90
	FG		50 − 27 − 19	3.80
		Parieto-occipital cortex	− 24 − 85 11	3.79
	Anterior STS/STG		60 3 − 21	3.79
		Middle STG/STS	− 54 − 23 − 2	3.74
		IFG	− 45 50 − 3	3.64
	Dorsal PFC		22 12 53	3.64
	Medial PFC		2 54 20	3.62
		Ventral tegmentum	− 2 − 17 − 22	3.60
	Lateral OFC		24 40 − 21	3.58
	Ventral tegmentum		5 − 10 − 5	3.50
		Medial PFC	− 4 59 36	3.48
	MTG		67 − 28 − 15	3.47
		MTG	− 65 − 9 − 22	3.42
	PHG		19 − 14 − 38	3.36
	Frontal pole		29 62 1	3.30
	Fornix		8 − 17 18	3.23
Faces**		IFG	− 50 40 − 4	4.08
		Anterior insula	− 36 15 4	3.96
	Anterior insula		29 26 − 4	3.85
		Lateral OFC	− 22 49 − 16	3.83
		Frontal operculum	− 37 28 − 9	3.81
Music > voices†		Lateral OFC	− 31 22 − 22	4.55
		Medial PFC	− 6 58 − 5	4.29
		Anterior insula	− 33 22 5	4.06
	Medial PFC		3 52 − 13	4.04
		Medial OFC	0 16 − 16	3.94

The Table shows maxima exceeding the specified threshold with an associated cluster extent of at least 50 voxels, derived from separate-modality regression analyses: *whole-brain correction based on false discovery rate p < 0.05; **small volume correction p < 0.05; † significantly stronger association with emotion recognition from music than voices based on a direct contrast in the combined-modalities analysis (small volume correction p < 0.05). No local maxima exceeded the specified threshold for the vocal modality-specific analysis, or for the direct comparisons between the music and face modalities in the combined-modalities analysis. Key: ACC, anterior cingulate gyrus; FG, fusiform gyrus; IFG, inferior frontal gyrus; MNI, Montreal Neurological Institute stereotactic space; MTG, middle temporal gyrus; OFC, orbitofrontal cortex; PFC, prefrontal cortex; PHG, parahippocampal gyrus; STG, superior temporal gyrus; STS, superior temporal sulcus.

## References

[bb0005] Adenzato M., Cavallo M., Enrici I. (2010). Theory of mind ability in the behavioural variant of frontotemporal dementia: an analysis of the neural, cognitive, and social levels. Neuropsychologia.

[bb0010] Adolphs R., Tranel D., Damasio H., Damasio A. (1994). Impaired recognition of emotion in facial expressions following bilateral damage to the human amygdala. Nature.

[bb0015] Anderson A.K., Spencer D.D., Fulbright R.K., Phelps E.A. (2000). Contribution of the anteromedial temporal lobes to the evaluation of facial emotion. Neuropsychology.

[bb0020] Baron-Cohen S., Wheelwright S., Hill J., Raste Y., Plumb I. (2001). The “Reading the Mind in the Eyes” test revised version: a study with normal adults, and adults with Asperger syndrome or high-functioning autism. J. Child Psychol. Psychiatry.

[bb0025] Baumgartner T., Lutz K., Schmidt C.F., Jäncke L. (2006). The emotional power of music: how music enhances the feeling of affective pictures. Brain Res..

[bb0030] Blood A.J., Zatorre R.J. (2001). Intensely pleasurable responses to music correlate with activity in brain regions implicated in reward and emotion. Proc Natl. Acad. Sci. U. S. A..

[bb0035] Boso M., Politi P., Barale F., Enzo E. (2006). Neurophysiology and neurobiology of the musical experience. Funct. Neurol..

[bb0040] Brown S., Martinez M.J., Parsons L.M. (2004). Passive music listening spontaneously engages limbic and paralimbic systems. Neuroreport.

[bb0045] Calder A.J., Lawrence A.D., Young A.W. (2001). Neuropsychology of fear and loathing. Nat. Rev. Neurosci..

[bb0050] Cardinal R.N., Parkinson J.A., Hall J., Everitt B.J. (2002). Emotion and motivation: the role of the amygdala, ventral striatum, and prefrontal cortex. Neurosci. Biobehav. Rev..

[bb0055] Carrington S.J., Bailey A.J. (2009). Are there theory of mind regions in the brain? A review of the neuroimaging literature. Hum. Brain Mapp..

[bb0060] Chan D., Fox N.C., Scahill R.I., Crum W.R., Whitwell J.L., Leschziner G., Rossor A.M., Stevens J.M., Cipolotti L., Rossor M.N. (2001). Patterns of temporal lobe atrophy in semantic dementia and Alzheimer's disease. Ann. Neurol..

[bb0065] Critchley H.D. (2009). Psychophysiology of neural, cognitive and affective integration: fMRI and autonomic indicants. Int. J. Psychophysiol..

[bb0070] Dolan R.J. (2007). The human amygdala and orbital prefrontal cortex in behavioural regulation. Philos. Trans. R. Soc. Lond. B Biol. Sci..

[bb0075] Drapeau J., Gosselin N., Gagnon L., Peretz I., Lorrain D. (2009). Emotional recognition from face, voice, and music in dementia of the Alzheimer type. Ann. NY Acad. Sci..

[bb0080] Edwards-Lee T., Miller B.L., Benson D.F., Cummings J.L., Russell G.L., Boone K., Mena I. (1997). The temporal variant of frontotemporal dementia. Brain.

[bb0085] Ekman P., Friesen W.V. (1976). Pictures of Facial Affect.

[bb0090] Eldar E., Ganor O., Admon R., Bleich A., Hendler T. (2007). Feeling the real world: limbic response to music depends on related content. Cereb. Cortex.

[bb0095] Eschrich S., Münte T.F., Altenmüller E.O. (2008). Unforgettable film music: the role of emotion in episodic long-term memory for music. BMC Neurosci..

[bb0105] Fernandez-Duque D., Black S.E. (2005). Impaired recognition of negative facial emotions in patients with frontotemporal dementia. Neuropsychologia.

[bb0110] Freeborough P.A., Fox N.C., Kitney R.I. (1997). Interactive algorithms for the segmentation and quantitation of 3-D MRI brain scans. Comput. Meth. Programs Biomed..

[bb0115] Fritz T., Jentschke S., Gosselin N., Sammler D., Peretz I., Turner R., Friederici A.D., Koelsch S. (2009). Universal recognition of three basic emotions in music. Curr. Biol..

[bb0120] Gagnon L., Peretz I., Fulop T. (2009). Musical structural determinants of emotional judgments in dementia of the Alzheimer type. Neuropsychology.

[bb0125] Gallagher H.L., Frith C.D. (2003). Functional imaging of ‘theory of mind’. Trends Cogn. Sci..

[bb0130] Genovese C.R., Lazar N.A., Nichols T. (2002). Thresholding of statistical maps in functional neuroimaging using the false discovery rate. Neuroimage.

[bb0135] Good C.D., Johnsrude I.S., Ashburner J., Henson R.N., Friston K.J., Frackowiak R.S. (2001). A voxel-based morphometric study of ageing in 465 normal adult human brains. Neuroimage.

[bb0140] Gosselin N., Peretz I., Noulhiane M., Hasboun D., Beckett C., Baulac M. (2005). Impaired recognition of scary music following unilateral temporal lobe excision. Brain.

[bb0145] Gosselin N., Samson S., Adolphs R., Noulhiane M., Roy M., Hasboun D., Baulac M., Peretz I. (2006). Emotional responses to unpleasant music correlates with damage to the parahippocampal cortex. Brain.

[bb0150] Gosselin N., Peretz I., Johnsen E., Adolphs R. (2007). Amygdala damage impairs emotion recognition from music. Neuropsychologia.

[bb0155] Griffiths T.D., Warren J.D., Dean J.L., Howard D. (2004). “When the feeling's gone”: a selective loss of musical emotion. J. Neurol. Neurosurg. Psychiatry.

[bb0160] Halpern A.R., Bartlett J.C., Dowling W.J. (1995). Aging and experience in the recognition of musical transpositions. Psychol. Aging.

[bb0165] Hanley J.A., McNeil B.J. (1982). The meaning and use of the area under a receiver operating characteristic (ROC) curve. Radiology.

[bb0170] Henley S.M., Wild E.J., Hobbs N.Z., Warren J.D., Frost C., Scahill R.I., Ridgway G.R., MacManus D.G., Barker R.A., Fox N.C., Tabrizi S.J. (2008). Defective emotion recognition in early HD is neuropsychologically and anatomically generic. Neuropsychologia.

[bb0175] Hillis A.E. (2007). Aphasia: progress in the last quarter of a century. Neurology.

[bb0180] Janes H., Pepe M.S. (2008). Adjusting for covariates in studies of diagnostic, screening, or prognostic markers: an old concept in a new setting. Am. J. Epidemiol..

[bb0185] Janes H., Longton G., Pepe M.S. (2009). Accommodating covariates in receiver operating characteristic analysis. Stata J..

[bb0190] Johnsen E.L., Tranel D., Lutgendorf S., Adolphs R. (2009). A neuroanatomical dissociation for emotion induced by music. Int. J. Psychophysiol..

[bb0195] Juslin P.N., Laukka P. (2003). Communication of emotions in vocal expression and music performance: different channels, same code?. Psychol. Bull..

[bb0200] Juslin P.N., Västfjäll D. (2008). Emotional responses to music: the need to consider underlying mechanisms. Behav. Brain Sci..

[bb0205] Keane J., Calder A.J., Hodges J.R., Young A.W. (2002). Face and emotion processing in frontal variant frontotemporal dementia. Neuropsychologia.

[bb0210] Kessels R.P., Gerritsen L., Montagne B., Ackl N., Diehl J., Danek A. (2007). Recognition of facial expressions of different emotional intensities in patients with frontotemporal lobar degeneration. Behav. Neurol..

[bb0215] Koelsch S. (2010). Towards a neural basis of music-evoked emotions. Trends Cogn. Sci..

[bb0220] Koelsch S., Fritz T., DY V.C., Muller K., Friederici A.D. (2006). Investigating emotion with music: an fMRI study. Hum. Brain Mapp..

[bb0225] Liu W., Miller B.L., Kramer J.H., Rankin K., Wyss-Coray C., Gearhart R., Phengrasamy L., Weiner M., Rosen H.J. (2004). Behavioral disorders in the frontal and temporal variants of frontotemporal dementia. Neurology.

[bb0230] Matthews B.R., Chang C.C., De May M., Engstrom J., Miller B.L. (2009). Pleasurable emotional response to music: a case of neurodegenerative generalized auditory agnosia. Neurocase.

[bb0235] Menon V., Levitin D.J. (2005). The rewards of music listening: response and physiological connectivity of the mesolimbic system. Neuroimage.

[bb0240] Mitterschiffthaler M.T., Fu C.H., Dalton J.A., Andrew C.M., Williams S.C. (2007). A functional MRI study of happy and sad affective states induced by classical music. Hum. Brain Mapp..

[bb0245] Molnar-Szakacs I., Overy K. (2006). Music and mirror neurons: from motion to ‘e'motion. Soc Cogn Aff Neurosci.

[bb0250] Neary D., Snowden J.S., Gustafson L., Passant U., Stuss D., Black S., Freedman M., Kertesz A., Robert P.H., Albert M., Boone K., Miller B.L., Cummings J., Benson D.F. (1998). Frontotemporal lobar degeneration: a consensus on clinical diagnostic criteria. Neurology.

[bb0255] Omar R., Hailstone J.C., Warren J.E., Crutch S.J., Warren J.D. (2010). The cognitive organisation of music knowledge: a clinical analysis. Brain.

[bb0260] Peretz I., Gagnon L., Bouchard B. (1998). Music and emotion: perceptual determinants, immediacy, and isolation after brain damage. Cognition.

[bb0265] Ridgway G.R., Omar R., Ourselin S., Hill D.L., Warren J.D., Fox N.C. (2009). Issues with threshold masking in voxel-based morphometry of atrophied brains. Neuroimage.

[bb0270] Rolls E.T. (2004). The functions of the orbitofrontal cortex. Brain Cogn..

[bb0275] Rosen H.J., Perry R.J., Murphy J., Kramer J.H., Mychack P., Schuff N., Weiner M., Levenson R.W., Miller B.L. (2002). Emotion comprehension in the temporal variant of frontotemporal dementia. Brain.

[bb0280] Rosen H.J., Pace-Savitsky K., Perry R.J., Kramer J.H., Miller B.L., Levenson R.W. (2004). Recognition of emotion in the frontal and temporal variants of frontotemporal dementia. Dement. Geriatr. Cogn. Disord..

[bb0285] Ross L.A., Olson I.R. (2010). Social cognition and the anterior temporal lobes. Neuroimage.

[bb0290] Salimpoor V.N., Benovoy M., Larcher K., Dagher A., Zatorre R.J. (2011). Anatomically distinct dopamine release during anticipation and experience of peak emotion to music. Nat. Neurosci..

[bb0295] Sauter D.A., Calder A.J., Eisner F., Scott S.K. (2010). Perceptual cues in non-verbal vocal expressions of emotion. Q. J. Exp. Psychol..

[bb0300] Saxe R., Carey S., Kanwisher N. (2004). Understanding other minds: linking developmental psychology and functional neuroimaging. Annu. Rev. Psychol..

[bb0305] Schroeter M.L., Raczka K., Neumann J., von Cramon D.Y. (2008). Neural networks in frontotemporal dementia—a meta-analysis. Neurobiol. Aging.

[bb0310] Seeley W.W., Carlin D.A., Allman J.M., Macedo M.N., Bush C., Miller B.L., Dearmond S.J. (2006). Early frontotemporal dementia targets neurons unique to apes and humans. Ann. Neurol..

[bb0315] Seeley W.W., Menon V., Schatzberg A.F., Keller J., Glover G.H., Kenna H., Reiss A.L., Greicius M.D. (2007). Dissociable intrinsic connectivity networks for salience processing and executive control. J. Neurosci..

[bb0320] Seeley W.W., Crawford R.K., Zhou J., Miller B.L., Greicius M.D. (2009). Neurodegenerative diseases target large-scale human brain networks. Neuron.

[bb0325] Snowden J.S., Bathgate D., Varma A., Blackshaw A., Gibbons Z.C., Neary D. (2001). Distinct behavioural profiles in frontotemporal dementia and semantic dementia. J. Neurol. Neurosurg. Psychiatry.

[bb0330] Snowden J.S., Austin N.A., Sembi S., Thompson J.C., Craufurd D., Neary D. (2008). Emotion recognition in Huntington's disease and frontotemporal dementia. Neuropsychologia.

[bb0335] Steinbeis N., Koelsch S. (2009). Understanding the intentions behind man-made products elicits neural activity in areas dedicated to mental state attribution. Cereb. Cortex.

[bb0360] Stewart L., von Kriegstein K., Warren J.D., Griffiths T.D. (2006). Music and the brain: disorders of musical listening. Brain.

[bb0340] Suzuki M., Okamura N., Kawachi Y., Tashiro M., Arao H., Hoshishiba T., Gyoba J., Yanai K. (2008). Discrete cortical regions associated with the musical beauty of major and minor chords. Cogn Aff Behav Neurosci.

[bb0345] Whitwell J.L., Crum W.R., Watt H.C., Fox N.C. (2001). Normalization of cerebral volumes by use of intracranial volume: implications for longitudinal quantitative MR imaging. AJNR Am. J. Neuroradiol..

[bb0350] Zentner M., Grandjean D., Scherer K.R. (2008). Emotions evoked by the sound of music: characterization, classification, and measurement. Emotion.

[bb0355] Zhou J., Greicius M.D., Gennatas E.D., Growdon M.E., Jang J.Y., Rabinovici G.D., Kramer J.H., Weiner M., Miller B.L., Seeley W.W. (2010). Divergent network connectivity changes in behavioural variant frontotemporal dementia and Alzheimer's disease. Brain.

