# Supplementary material for: The structural neuroanatomy of music emotion recognition: Evidence from frontotemporal lobar degeneration
Source: Neuroimage. 2011 Jun 1;56(3):1814–21. doi: 10.1016/j.neuroimage.2011.03.002 (PMC3092986; doi:10.1016/j.neuroimage.2011.03.002)
Supplement: Supplementary file 1 — Supplementary materials [file mmc1.pdf]

SUPPLEMENTARY MATERIAL

Experimental battery for assessment of emotion recognition in music, faces and voices

Stimuli: music

The stimuli for recognition of emotion in music were excerpts drawn from the Western classical canon and film scores (mean duration (range) as follows: anger 11.6 sec (9.8 – 13.3); fear, 12.2 sec (10.3 – 16.4); happiness, 10.5 sec (8 – 13.3); sadness, 11.6 sec (10.1 – 16)). Most pieces were orchestral works; some chamber pieces were also included. No vocal musical excerpts were included. Stimuli are listed in Supplementary Table 1.

Selection of stimuli was based on an initial pilot study in 16 healthy subjects who did not participate in the subsequent experiment. Pilot subjects were presented with a larger set of 104 musical excerpts and asked to rate each excerpt for how strongly it represented each of the four target emotions using a paper scale ranging from 0 (not at all) to 4 (very strongly). Ratings for each excerpt for each emotion were averaged across the control group. An excerpt for which one and only one emotion achieved a mean rating ≥ 2 was considered to portray that emotion (other excerpts were considered insufficiently salient, or ambiguous). Excerpts fulfilling this criterion were ordered based on rating, and the 10 highest-ranking excerpts for each emotion were used in the test battery. Mean (range) ratings for each emotion were as follows: anger, 3.0 (2.8 – 3.8); fear, 3.1 (2.5 – 3.8); happiness, 3.2 (2.6 – 3.9); sadness, 2.8 (2.1 – 3.5).

Stimuli: facial expressions

The facial emotion stimuli comprised black and white photographs of posed facial expressions derived from the set produced by Ekman and Friesen (1976); the most reliably recognised exemplars from the original set for each target emotion were selected.

Stimuli: nonverbal vocal sounds

The vocal emotion stimuli were brief nonverbal vocalisations recorded by male and female actors to express each of the same target canonical emotions (Sauter et al., 2010). The most reliably recognised exemplars from the original set for each target emotion were selected.

General testing procedure

Auditory stimuli were presented as digital wavefiles on a notebook computer in free field at a comfortable listening level in a quiet room. Visual stimuli were presented and subject responses were collected for off-line analysis in Cogent 2000 (www.vislab.ucl.ac.uk/Cogent2000) running under MATLAB 7.0® (http://www.mathworks.com). For each modality, 40 trials were presented, comprising 10 stimuli representing each of the four target canonical emotions. Modalities were presented in a block design, in the order: faces, vocal sounds, music. Within each modality (block), the 40 trials were presented in pseudo-randomised order (i.e., for a particular subject the order of stimulus presentation was random but this same order was used for all subjects). On each trial, the subject was asked to choose which one of the four target emotions was best represented by the stimulus. The words corresponding to the choices on each trial were simultaneously displayed on the computer monitor and spoken by the examiner. Before the start of each modality block, four practice trials were administered to ensure the subject understood the task. No feedback about performance was given during the test. No time limit was imposed. Subject responses were stored for off-line analysis.

Supplementary Figure 1. Statistical parametric map (SPM) of grey matter loss associated with impaired emotion recognition from music in FTLD: effect of covarying for general executive performance (Trails score). The SPM is thresholded at $p<0.05$ FDR corrected for multiple comparisons over the whole brain volume and presented on sections of the mean normalised T1-weighted structural brain image in MNI stereotactic space; the left hemisphere is on the left and slice coordinates in mm are shown. Letter codes are as for Figure 2.

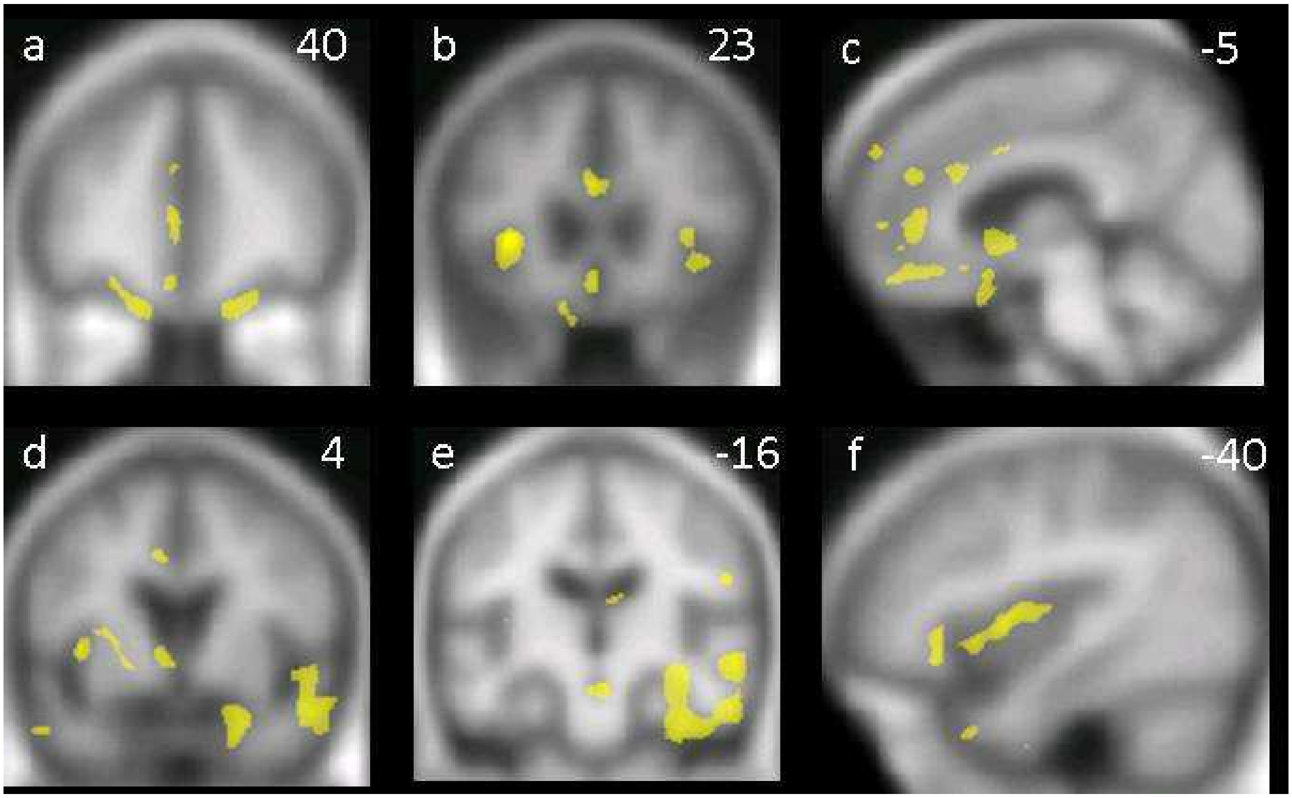

Supplementary Table 1. Stimuli used to represent canonical emotions in music

Anger
Egmont Overture (Beethoven)
Enigma Variation No. 4: Allegro di molto (Elgar)
Mars, from The Planets (Holst)
New World Symphony: Allegro (Dvorak)
New World Symphony: Scherzo (Dvorak)
Organ Symphony: Scherzo (Saint Saens)
Summer, from The Four Seasons (Vivaldi)
Symphony No. 5: Moderato (Shostakovich)
Symphony No. 5: Allegro non troppo (Shostakovich)
Symphony No. 6: Storm (Beethoven)

Fear
Aliens Theme
Alien 3 Theme
Bluebeard's Castle: The Lake of Tears (Bartok)
Concerto Grosso No 3 for Two Violins and Harpsichord: Pesante (Schnittke)
Jaws Theme
Music for Strings, Percussion and Celesta: Adagio (Bartok)
Night on a Bare Mountain (Mussorgsky)
Pictures at an Exhibition: Cum Mortuis (Mussorgsky)
Psycho Theme
Saturn, from The Planets (Holst)

Happiness
Autumn, from The Four Seasons (Vivaldi)
Big Country Theme
La Boheme Overture (Puccini)
Canon in D (Pachelbel)
Capriccio Espagnol: Alborada (Rimsky-Korsakov)
Capriccio Espagnol: Fandango (Rimsky-Korsakov)
Jurassic Park Theme
Marriage of Figaro Overture (Mozart)
Ma Vlast: Vltava (Smetana)
Romanze in F (Brahms)

Sadness
Adagio for Strings (Barber)
La Boheme Finale (Puccini)
Fantasia on a Theme by Thomas Tallis (Vaughan Williams)
Intermezzo in A major, Opus 118 (Brahms)
Pathetique Sonata: Grave (Beethoven)
Russian Easter Festival Overture (Rimsky-Korsakov)
Scheherazade (Rimsky-Korsakov)
Schindler's List Theme
Symphony No 3: Poco Allegretto (Brahms)
Symphony No. 5: Largo (Shostakovich)

Supplementary Table 2 Mean scores for healthy control, bvFTD and SemD groups on tests of emotion recognition in different modalities and for individual emotion:modality combinations.

Modality	Emotion	Mean score (SD) (95% CI for mean)		
		**Controls n=21**	**bvFTD n=16**	**SemD n=10**
Music	Total /40	32.9 (2.63) (31.8, 34.0)	21.8 (5.55) (19.2, 24.4)	21.2 (6.03) (17.6, 24.7)
	Happiness /10	8.86 (1.24) (8.33, 9.38)	7.81 (1.94) (6.81, 8.63)	7.10 (1.73) (6.20, 8.20)
	Sadness /10	9.29 (1.01) (8.86, 9.67)	6.81 (2.48) (5.56, 7.94)	6.40 (1.96) (5.10, 7.40)
	Anger /10	7.38 (1.60) (6.76, 8.10)	3.13 (1.75) (2.31, 4.00)	3.20 (1.93) (1.90, 4.20)
	Fear /10	7.38 (1.16) (6.95, 7.90)	4.06 (1.95) (3.19, 5.06)	4.50 (2.51) (3.10, 6.00)
Faces	Total / 40	37.6 (1.40) (37.0, 38.1)	32.3 (4.29) (30.2, 34.3)	32.5 (5.87) (28.5, 35.4)
	Happiness /10	10 (0)*	9.75 (0.58) (9.50, 10.0)	9.90 (0.32) (9.80, 10.0)
	Sadness /10	9.62 (0.81) (9.33, 10.00)	8.50 (1.46) (7.81, 9.19)	8.20 (1.93) (7.00, 9.20)
	Anger /10	8.14 (1.15) (7.62, 8.57)	6.56 (2.10) (5.63, 7.56)	6.90 (2.03) (5.50, 7.90)
	Fear /10	9.86 (0.48) (9.67, 10.00)	7.50 (2.00) (6.56, 8.44)	7.50 (2.22) (6.20, 8.80)
Voices	Total /40	35.0 (3.26) (33.4, 36.1)	29.7 (5.85) (26.7, 32.3)	29.0 (8.21) (23.8, 33.4)
	Happiness /10	8.24 (1.61) (7.43, 8.81)	8.25 (1.77) (7.44, 9.06)	7.70 (2.26) (6.30, 9.00)
	Sadness /10	9.43 (0.87) (8.95, 9.71)	8.13 (2.19) (7.00, 9.06)	7.30 (2.00) (6.00, 8.40)
	Anger /10	8.24 (1.51) (7.52, 8.81)	6.75 (1.91) (5.94, 7.81)	6.90 (2.51) (5.20, 8.20)
	Fear /10	9.05 (1.32) (8.48, 9.57)	6.56 (2.63) (5.38, 7.81)	7.10 (3.07) (5.20, 8.80)

CI, confidence interval; SD, standard deviation; * CI not reported: since all controls scored 10/10 for recognition of happy faces, bootstrapping cannot provide a valid CI.

Supplementary Table 3. Estimated areas under the covariate (age, gender, years of education) adjusted ROC curves (95% bootstrap CI) for emotion modalities and emotion-modality combinations, comparing FTLD patients with healthy control subjects and FTLD subgroups

**Modality**	**Emotion**	**Differences between groups: expressed as areas under the adjusted* ROC curves for discriminating between groups (95% CI)**			
		**FTLD vs controls**	**bvFTD vs controls**	**SemD vs controls**	**SemD vs bvFTD**
Music	Total /40	**0.98 (0.86, 1)**	**0.98 (0.78, 1)**	**0.97 (0.83, 1)**	0.47 (0.22, 0.73)
	Happiness /10	0.70 (0.49, 0.85)	0.63 (0.37, 0.84)	**0.81 (0.56, 0.94)**	0.30 (0.10, 0.56)
	Sadness /10	**0.88 (0.76, 0.97)**	**0.87 (0.71, 0.98)**	**0.90 (0.68, 1)**	0.45 (0.21, 0.72)
	Anger /10	**0.97 (0.90, 1)**	**0.98 (0.89, 1)**	**0.97 (0.83, 1)**	0.53 (0.24, 0.83)
	Fear /10	**0.92 (0.81, 0.99)**	**0.98 (0.89, 1)**	**0.83 (0.57, 1)**	0.64 (0.39, 0.87)
Faces	Total / 40	**0.95 (0.84, 0.99)**	**0.98 (0.87, 1)**	**0.90 (0.69, 1)**	0.61 (0.36, 0.87)
	Happiness /10	**0.58 (0.54, 0.67)****	**0.59 (0.53, 0.75)****	**0.55 (0.50, 0.75)****	0.55 (0.41, 0.69)**
	Sadness /10	**0.78 (0.62, 0.91)**	**0.79 (0.54, 0.95)**	0.76 (0.47, 0.97)	0.52 (0.26, 0.83)
	Anger /10	**0.73 (0.56, 0.86)**	**0.77 (0.56, 0.93)**	0.67 (0.39, 0.88)	0.56 (0.31, 0.81)
	Fear /10	**0.93 (0.84, 0.99)**	**0.96 (0.88, 1)**	**0.89 (0.66, 1)**	0.54 (0.31, 0.80)
Voices	Total /40	**0.76 (0.58, 0.91)**	0.71 (0.46, 0.90)	**0.84 (0.60, 0.99)**	0.35 (0.14, 0.62)
	Happiness /10	0.45 (0.27, 0.64)	0.35 (0.15, 0.60)	0.61 (0.35, 0.86)	0.27 (0.08, 0.51)
	Sadness /10	**0.74 (0.54, 0.90)**	0.68 (0.41, 0.89)	**0.83 (0.58, 0.99)**	0.33 (0.13, 0.60)
	Anger /10	**0.68 (0.51, 0.84)**	0.63 (0.41, 0.85)	0.75 (0.49, 0.95)	0.43 (0.21, 0.72)
	Fear /10	**0.76 (0.60, 0.88)**	**0.76 (0.55, 0.91)**	0.75 (0.48, 0.95)	0.51 (0.27, 0.78)

*areas adjusted for age, gender, and years of education; ** area under the unadjusted ROC curve shown, since covariate effects in controls could not be estimated due to all controls scoring 10/10 for happy faces. bvFTD, behavioural variant frontotemporal dementia; CI, confidence interval; FTLD, frontotemporal lobar degeneration; SD, standard deviation; SemD, semantic dementia. Confidence intervals excluding 0.5 (**bold**) provide evidence that the corresponding measure has statistically significant discriminatory power for that comparison.

Supplementary references

Ekman P, Friesen WV. Pictures of facial affect. Palo Alto CA, Consulting Psychologists Press, 1976.

Sauter DA, Calder AJ, Eisner F, Scott SK. Perceptual cues in non-verbal vocal expressions of emotion. Q J Exp Psychol 2010; Apr 28:1-22
